# The Relationship Between Smartphone Overuse and Academic Achievement Among Undergraduate Nursing Students

**DOI:** 10.7759/cureus.48340

**Published:** 2023-11-06

**Authors:** Eman Bajamal, Shahrazad M Timraz, Sharifa Al syed, Erada Bajbeir, Wafaa BinAli

**Affiliations:** 1 Community Health Nursing, College of Nursing, King Saud bin Abdulaziz University for Health Sciences, Jeddah, SAU; 2 Nursing, College of Nursing, King Saud bin Abdulaziz University for Health Sciences, Jeddah, SAU; 3 Health Policy, College of Nursing, King Saud bin Abdulaziz University for Health Sciences, Jeddah, SAU; 4 Critical Care, College of Nursing, King Saud bin Abdulaziz University for Health Sciences, Jeddah, SAU

**Keywords:** education, undergraduate students, smartphone overuse, saudi arabia, academic achievement

## Abstract

Introduction

Nowadays, college students highly depend on smartphones on a daily basis because it is found to be practical and useful to manage and organize most of their daily basic activities. However, this raises a concern about the negative influence of smartphone overuse on their academic achievement and well-being. Relatively, multiple studies have reported negative impacts associated with smartphone overuse on different aspects like sleep patterns, body energy, eating habits, and academic achievements.

Objective

This cross-sectional study examines the relationship between smartphone overuse and academic achievement among 133 undergraduate nursing students at King Saud bin Abdulaziz University for Health Science in the Kingdom of Saudi Arabia.

Method

Demographic data was collected and the Smartphone Addiction Scale-Short Version (SAS-SV) was used to collect other data.

Results

The statistics showed an overuse of smartphones among the students as half of them (50.4%) reported checking their smartphones 1-10 times per hour. Additionally, most of the participants reported that they use their smartphones regularly (97.7%) and 90.2% of them use monthly data plans. Furthermore, 75.9% of students reported using their phones during class, either for note-taking or searching.

Conclusion

The findings did not show a significant association between academic achievement and smartphone overuse because a high percentage of the participants reported using their phones mostly for educational purposes.

## Introduction

The 21st century has witnessed major technological changes since the introduction of mobile phone technology in 1983 [1]. With the advancement in technology, mobile phones have evolved, and in 2009 smartphones were introduced to the market [2]. Since then, smartphones have become an essential part of an individual’s daily life. In 2017, smartphones were used by 2.32 billion; the rise in the use of smartphones was expected to reach 2.87 billion by 2020 [3]. The high prevalence of smartphone use is not surprising considering their substantial influence on improving and simplifying life. The various functions and features of this pocket-sized gadget including wireless 24/7 internet connection, keyboard, high-resolution camera, and large storage capacity allow them to be used for communication, gaming, social networking, and studying [4]. Despite the usefulness of smartphone, people become more dependent on them in everyday functioning [5], which escalate the concern of their negative influence on individual’s well-being. Several studies reported that smartphone overuse is associated with negative impacts on levels of energy, sleep, eating behaviors, body weight, exercise, and academic achievements [6,7]. This study will only focus on smartphone use and academic performance. Thus, the aim of this study is to determine the relationship between smartphone overuse and academic achievement among undergraduate nursing students at King Saud bin Abdulaziz University for Health Science in the Kingdom of Saudi Arabia.

Smartphones have multiple advantages in educational settings such as participating in active learning, accessing educational material, and browsing and sharing information with others [2], which may account for the large number of smartphone use among college students [6,8]. Furthermore, the availability of electronic resources with convenient access is shown to facilitate the learning process among students [9]. Despite these advantages, it was found that problematic smartphone use (PSU) negatively impacts academic performance among college students [2,9,10]. Problematic smartphone use (PSU) is defined as excessive use of smartphones associated with some criteria found in the contemporary addiction models such as dependence, withdrawal, and functional impairment [2].

Although research studies about the impact of smartphones on academic achievement are scarce, some studies concluded a positive association between smartphones and academic achievement [2,10]. For instance, a study of 293 undergraduate university students that aimed to verify if low Grade Point Averages (GPAs) are likely among students at high risk of smartphone addiction found that students who were at high risk of smartphone addiction were less likely to achieve cumulative GPAs of distinction or higher [10]. In addition, a study aimed to investigate the impact of smartphone use by college students on their perceived academic performance concluded that college students with higher smartphone self-efficacy (i.e., perceived ease of use and usefulness of smartphone) were more likely to anticipate improvement in their academic performance [11]. On the other hand, a study conducted [12] to examine the relationship between academic performance and Facebook usage among 219 university graduate and undergraduate students found that Facebook users had lower GPAs and reported less time for studying compared to their counterparts who did not use social networking services (SNS). Furthermore, only 26% of students reported the positive impact of SNS on their performance such as using FB to form groups. On the other hand, 74% of students reported negative impacts, such as procrastination, distraction, and time management. Lastly, a cross-sectional study of 181 medical undergraduate students in Saudi Arabia that aimed to examine smartphone addiction and determining factors for addiction, concluded that 36.5% of students were smartphone addicts [13]. Furthermore, 55% of smartphone addict reported using their phones more than five hours a day [13].

The aforementioned studies showed the high prevalence of smartphone use among college students and its negative impact on their academic performance. Nonetheless, there is a paucity of research in this area among undergraduate college students in the Kingdom of Saudi Arabia taking into account that Saudi Arabia has been ranked third in the world in terms of the population using smartphones because almost half of its population is below the age of 30 years [14]. Therefore, the aim of this study is to determine the relationship between smartphone overuse and academic achievement among undergraduate nursing students at King Saud bin Abdulaziz University for Health Science in the Kingdom of Saudi Arabia.

## Materials and methods

Design and setting

This cross-sectional study was conducted at King Saud bin Abdulaziz University for Health Sciences, College of Nursing-Jeddah (KSAU-HS, CON-J), among female nursing students who were enrolled in academic levels 5 to 8. The convenience sample technique was used to collect 133 participants. The participants were recruited virtually via e-mails, blackboard, and Microsoft Teams (Microsoft Corporation, Redmond, USA).

All participants included in the study were: (1) female students; (2) enrolled in the College of Nursing-Jeddah; and (3) voluntarily participating. Institutional Review Board approval (IRB) was obtained from King Abdullah International Medical Research Center (KAIMRC) with approval number RJ20/161/J. All students were invited to participate in the study; however, participation was voluntary, and the sampling process was continued until the fulfillment of the sample size from each level.

Sample size

The total number of participants in this study was 133. The sample size was estimated by G*Power software (Dusseldorf University, Germany), which allows sample size analysis and high-precision power and computes the power values for sample size, medium effect size, and alpha level. The aim was to include n = 119 to achieve the power of 85% with a medium effect size = 0.3, an error probability = 15%, and missing data estimated at 10%. The total sample size was N = (119+14) = 133.

Data collection methods

Data were collected using the previously tested and validated tool, Smartphone Addiction Scale Short Version (SAS-SV). In addition, a demographic questionnaire developed by the authors (age, educational level, parents’ education level, family monthly income, GPA) was used. The questionnaire was sent to students through email, blackboard, and Microsoft Teams.

Measures

Smartphone use was assessed by the English version of SAS-SV developed by Kwon et al. (2013) to measure smartphone overuse. It consists of a six-point Likert-type scale that ranges from 1 to 6; (1 = strongly disagree to 6 = strongly agree) based on self-reporting, with 33 items and six subscales (daily-life disturbance, positive anticipation, withdrawal, cyberspace-oriented relationship, overuse, and tolerance). The Cronbach alpha of the tool was a = .77 based on the study conducted by Kwon [15].

Kwon et al. (2013) described the SAS subscales as follows: “Daily-life disturbance” describes the difficulty of concentrating in class, missing planned work, and suffering from disturbances, such as lightheadedness, sleep, or neck pain. “Positive anticipation” describes reducing stress by using the smartphone and feeling empty when there is no smartphone. “Withdrawal” describes the impatient, fretful, and intolerable feelings when there is no smartphone. “Cyberspace-oriented relationship” describes closer relationships with friends on social networking services than in real life. “Overuse” describes uncontrollable smartphone use. “Tolerance” can be described as always trying to control the use of the smartphone, but not being successful [15].

A higher SAS score indicates a more serious smartphone addiction. Total SAS score among study participants ranged between 10-56 (minimum and maximum). Internal consistency of the SAS used among study participants was acceptable (a = .77).

The students were categorized into three groups based on their GPA: Group A (GPA 4.75 to 5.00); Group B (GPA 4.00 to 4.75), and Group C (GPA 3.00 to 3.99).

Statistical analysis

The Statistical Package for the Social Sciences computer software (SPSS 20, IBM Corp., Armonk, USA) was used to analyze the data presented in this study. Descriptive statistics were expressed as mean ± standard deviation (SD), frequency, and percentage. To assess the normality of the total SAS score, the Shapiro-Wilk test was used and the test showed that the distribution was normal (p = 0.745). Student's t-test and ANOVA were used to compare the mean GPA and total SAS score across the different groups (academic level, marital status, having children, etc.). A multiple linear regression model was used to assess the association between the total SAS score (predictor) and the college performance expressed by last semester's GPA (outcome), adjusted for academic level. Statistical significance was based on the standard alpha level of .05.

## Results

Demographic characteristics

A total of 133 participants were included in the final analysis after excluding two participants due to missing data (1.48%). As shown in Table 1, the largest percentage of the participants was from level 6 who represented the largest number of all levels for that academic year. Participants’ age ranged from 20 to 24 years with a mean of 21.7 ± 1.04 years. Thirty-seven percent of participants were from level 6 (n = 50), while the rest were from other levels (n = 85, 63%). The majority of the participants were single (n = 123, 91.1%), while only 11% of them were married and 0.7% were divorced. Approximately 94.1% of the participants did not have children while only 4.4% of them had children. Most of the participants (n = 120, 88.9%) were living with their parents, while the others were living with their husband and children (n = 6, 4.4%), with other family members (n = 7, 5.2%), or alone (n = 2, 1.5%).

Parents' education level ranged from uneducated to undergraduate degree or higher. As shown in Table 1, the largest percentage of participants' father's education was up to high school (n = 40, 29.6%), compared with fathers who had an undergraduate degree (n = 28, 20.7%). For mother's education, the largest percentage reflected having an undergraduate degree (n = 41, 30.4%), compared with mothers who had passed high school (n = 30, 22.2%).

**Table 1:** Sample demographic characteristics (N = 133) * US$1 = SR3.75

Demographic variable	n	%	
Academic level			
Level 5	26	19.3	
Level 6	50	37.0	
Level 7	23	17.0	
Level 8	36	26.7	
Academic performance			
A	8	6.00	
B	81	60.9	
C	44	33.1	
Marital status			
Single	123	91.1	
Married	11	8.30	
Divorced	1	0.80	
Having children			
Yes	6	4.50	
No	127	95.5	
Living with whom			
Parents	120	88.9	
Other family members	7	5.20	
Husband and/or children	6	4.40	
Alone	2	1.50	
Father’s education level			
Not educated	5	3.70	
Elementary School	17	12.6	
Intermediate School	22	16.3	
High School	40	29.6	
Associate degree	14	10.4	
Undergraduate degree	28	20.7	
Graduate degrees (Master or PhD)	9	6.70	
Mother’s education level			
Not educated	7	5.20	
Elementary School	19	14.1	
Intermediate School	21	15.6	
High School	30	22.2	
Associate degree	14	10.4	
Undergraduate degree	41	30.4	
Graduate degrees (Master or PhD)	3	2.20	
Family monthly income in SR*			
< 3000	11	8.10	
3000-7000	37	27.4	
7000-12000	29	21.5	
12000-17000	21	15.6	
≥ 17000	33	24.4	

For the family income, as shown in Table 1, the largest percentage was associated with having a family income between Saudi Riyal (SR) 3,000 and SR7,000 (n = 37, 27.4%), whereas the lowest percentage was reported for the participants whose family income was less than SR3,000 (n = 11, 8.1%). 60.9% (n = 81) of participants reported a GPA within B grade (3.81 ± 0.56) in the last semester. A detailed description of the characteristics of the study participants is presented in Table 1.

Smartphone overuse

A detailed description of smartphone overuse among participants included in the study is provided in Table 2. Data show that 91.0% (n = 121) of participants used smartphones during weekdays and on weekends. The majority of the participants reported using smartphones regularly (n = 130, 97.7%). Around 94% of participants use iPhones (n = 125), whereas 90.2% (n = 120) of participants reported having a data plan. Most of the participants (n = 104, 77.0%) reported using smartphones when studying, while 75.9% (n = 101) reported using smartphones to text in class. Half of the participants (n = 67, 50.4%) reported checking their smartphones 1-10 times per hour. The mean total SAS score was 34.3 ± 8.90. 

**Table 2:** Smartphone overuse in study participants (N = 133).

Variables	n	%
Time of smartphone use		
Weekdays and weekend	121	91.0
Weekdays	9	6.80
Weekend	3	2.30
Regular use of smartphone		
Yes	130	97.7
No	3	2.30
Type of cell phone		
iPhone	125	94.0
Samsung	6	4.50
Others	2	1.50
Having data plan with smartphone		
Yes	120	90.2
No	13	9.80
Using smartphone when studying		
Yes	104	77.0
No	27	20.0
Texting in class		
Always	16	12.0
Sometimes	85	63.9
Never	32	24.0
Checking smartphone per hour		
1-10 times	66	50.4
11-20 times	19	14.3
20-30 times	23	17.3
30-40 times	10	7.50
> 40 times	14	10.5

Association between the characteristics of participants, academic performance, and smartphone overuse

The association between the characteristics of participants, academic performance, and smartphone overuse is presented in Table 3. Data show that academic level was significantly associated with academic performance (p = 0.001), where the mean score of students’ GPAs was significantly higher among students in level 6 compared to students in level 7, whereas the mean GPA score and total SAS score were similar across the different participants’ levels.

**Table 3:** Association between characteristics of participants, academic performance, and smartphone overuse (N = 133) The numbers presented in the table are means ± SDs. * Significant at the 95% confidence level; SAS: Smartphone Addiction Scale

Characteristics of Participants	GPA	Total SAS score
Academic level		
Level 5	3.64 ± 0.56	33.6 ± 9.62
Level 6	4.01 ± 0.60	34.9 ± 9.22
Level 7	3.45 ± 0.47	35.4 ± 8.69
Level 8	3.88 ± 0.43	33.3 ± 8.55
p-value	0.001*	0.807
Marital status		
Single	3.82 ± 0.56	34.3 ± 8.95
Married	3.59 ± 0.52	35.1 ± 9.58
Divorce	n/a	n/a
p-value	0.283	0.965
Having children		
Yes	3.50 ± 0.58	33.0 ± 8.98
No	3.82 ± 0.56	33.4 ± 8.94
p-value	0.266	0.765
Living with whom		
Parents	3.82 ± 0.55	34.0 ± 8.81
Other family members	3.94 ± 0.81	42.0 ± 7.52
Husband and/or children	3.60 ± 0.55	35.8 ± 11.8
Alone	3.50 ± 0.71	29.0 ± 2.83
p-value	0.664	0.197
Father’s education level		
< High school	3.74 ± 0.58	34.6 ± 7.85
High-school/Diploma	3.86 ± 0.53	34.0 ± 10.2
≥ University degree	3.80 ± 0.58	34.5 ± 8.16
p-value	0.613	0.960
Mother’s education level		
< High school	3.77 ± 0.60	35.0 ± 6.88
High school/Diploma	3.70 ± 0.52	34.1 ± 9.52
≥ University degree	3.96 ± 0.53	33.9 ± 10.2
p-value	0.110	0.845
Family monthly income in SR		
< 3000	3.51 ± 0.64	37.6 ± 5.87
3000-7000	3.67 ± 0.58	34.0 ± 8.96
7000-12000	3.91 ± 0.42	33.3 ± 7.28
12000-17000	4.04 ± 0.70	32.6 ± 10.6
≥ 17000	3.79 ± 0.47	35.4 ± 10.0
p-value	0.068	0.595

Association between smartphone use, academic performance, and total SAS score

As shown in Table 4, the data show that GPA was significantly associated with texting in class, where the mean score of GPAs was significantly low among the participants who always text in class (p = .001), whereas the mean GPA score was not statistically significant across all other groups. Moreover, the total SAS score was associated with the time of smartphone use and regular use of the smartphone, where a significantly higher mean total SAS score was found among the participants who only used the smartphone during the weekend (p = .006). In addition, a significantly high mean total SAS score was found among the participants who reported regular use of the smartphone compared to the participants who did not use the smartphone regularly (34.7 ± 8.61 vs. 16.5 ± 6.36, respectively, p = 0.004). 

**Table 4:** Association between smartphone use, academic performance, and total SAS score (N = 133) The numbers presented in the table are mean ± SD. * Significant at 95% confidence level.

Characteristics of Participants	GPA	Total SAS score
Time of smartphone use		
Weekdays and weekend	3.81 ± 0.56	34.7 ± 8.08
Weekdays	3.91 ± 0.55	27.0 ± 12.6
Weekend	3.00 ± 0.00	46.5 ± 12.0
p-value	0.306	0.006*
Regular use of smartphone use		
Yes	3.80 ± 0.56	34.7 ± 8.61
No	4.05 ± 0.64	16.5 ± 6.36
p-value	0.534	0.004*
Type of cell phone		
iPhone	3.81 ± 0.56	34.1 ± 8.66
Samsung	3.68 ± 0.70	39.5 ± 11.7
Others	3.75 ± 0.35	23.0 ± 0.00
p-value	0.866	0.158
Having data plan with smartphone		
Yes	3.83 ± 0.54	34.3 ± 9.20
No	3.53 ± 0.65	34.1 ± 4.81
p-value	0.089	0.943
Using smartphone when studying		
Yes	3.78 ± 0.56	34.6 ± 8.69
No	3.92 ± 0.53	33.3 ± 9.79
p-value	0.304	0.560
Texting in class		
Always	3.42 ± 0.44	36.6 ± 14.1
Sometimes	3.95 ± 0.53	34.5 ± 7.51
Never	3.66 ± 0.57	32.5 ± 9.51
p-value	0.001*	0.400
Checking smartphone per hour		
1-10 times	3.73 ± 0.58	34.2 ± 8.96
11-20 times	3.96 ± 0.61	35.4 ± 8.50
21-30 times	3.94 ± 0.42	34.1 ± 6.38
31-40 times	3.83 ± 0.43	35.5 ± 9.97
> 40 times	3.73 ± 0.59	31.9 ± 13.2
p-value	0.453	0.902

Association between smartphone overuse and academic performance

The total SAS mean score was similar across the different groups based on the academic performance in the last semester (A: 37.1 ± 6.50; B: 33.8 ± 8.62; C: 34.7 ± 9.88, p = 0.605).

Multiple linear regression model to investigate the association between total SAS score and academic performance (last semester GPA), adjusted for academic level, show no association between total SAS score and academic performance (B = -0.004, SE= .006 [95% Confidence Interval: -0.02 to 0.01], p = 0.513, R-square= 0.01).

Table 5 demonstrates Pearson’s product-moment correlation among the study variables. As shown in Table 5, age was statistically and negatively correlated with the GPA (r = -.193, p < .05), and negatively and weakly correlated with SAS (r = -.033, p < .05). The GPA was negatively and weakly correlated with SAS (r = -.060, p < .05). This weak association could be due to the small sample size for the descriptive study.

**Table 5:** Pearson’s product-moment correlations between age, GPA, and SAS (N = 133) *p < .05 SAS: Smartphone Addiction Scale; GPA: Grade Point Average

Variable	1	2	3
1- Age in years	1		
2- Recent GPA	-.193*	1	
3- SAS Total	-.033	-.060	1

## Discussion

This study determined the relationship between smartphone overuse and academic achievement among 133 undergraduate nursing students at KSAU-HS, Jeddah, KSA. Based on the results, no significant relationship was found between the demographic characteristics (i.e., academic level, marital status, parent’s education level, social income, etc), GPA, and smartphone overuse. This result is congruent with another study conducted by Sulaiman and Alebrahim [16], in which no significant difference was found between problematic smartphone overuse and demographic characteristics. On the contrary, a study conducted by Aljomaa et al., found a significant relationship between problematic smartphone use and marital status [17], which is also congruent with the finding of Vaziri-Harami et al. [18]. Nonetheless, the study by Aljomma et al. included 50.96% male and 49.38% female participants across different academic levels (i.e., undergraduate and graduate) and specialties [17]. However, the current study only included undergraduate nursing students at four different academic levels which might be counted for the differences in the findings.

Regarding the academic level (i.e., year of study), a significant relationship was found between the academic level and GPA, but not smartphone overuse in the current study. Nonetheless, the study by Sulaiman and Alebrahim, reported that junior students were more addicted to smartphone use compared to senior students [16]. However, their study included students from different specialties, contrary to this study, which only included nursing students. Furthermore, the significant association between academic level and GPA in the current study might be attributed to the number and types of courses given at level 6 and their difficulty. In other words, students at the entry level are introduced for the first time to foundational nursing courses and at the advanced levels (i.e., 7 and 8) to specialized nursing courses that have clinical components. Level 6 is considered the transition level between the entry and advanced ones.

Family income was not associated with GPA or smartphone overuse in the current study. A similar finding is found in Aljomma et al. in which smartphone addiction was not associated with the economic status of the participants [17]. On the contrary, multiple studies reported that students from high-income families tended to use their phones more frequently [19,20]. It is worth mentioning that the participants in this study received a monthly stipend from the university which they may use to purchase a smartphone or have a data plan irrespective of the economic status of their family. Additionally, more than half of the participants were living with their parents with a monthly income of more than SR7,000.

Parents’ educational level also was not significantly associated with GPA or smartphone overuse, which is consistent with a study by Ahn (2011) that concluded parents' education was not a significant predictor of using social network sites among teens [21].

A smartphone is a small gadget that can be carried everywhere and anywhere offering a variety of tools and applications to navigate through and to be used for education, communication, and socialization purposes. Similarly, the current study shows that most students use their smartphones regularly on weekends and weekdays as the majority of them are using monthly data plans, and this is consistent with the literature [22], taking into account college students’ fascination with using technology. A comparative study of cell phone use among college students from the US and India reported that participants agreed that having a data plan affected their usage of cell phones [23]. A significant relationship was found between the regular use of smartphones and the total SAS, which is expected because regular use can put students at risk for smartphone addiction. Furthermore, the total SAS score was significantly associated with the time of smartphone use (p = 0.006). A similar finding was seen in another study conducted among Kuwaiti college students in which smartphone addiction was significantly associated with the time spent on smartphones [16]. 

No relationship was found in the current study between smartphone overuse and academic achievements. Similar findings were reported by other studies [3,16,24]. For instance, a study conducted by Alsayed et al. (2020) to evaluate the practice of smartphone use among undergraduate nursing students found a high frequency of smartphone application use among nursing students for educational purposes as they frequently access scientific websites to find related medical information as well as using their phones to check academic announcements via emails and receive courses related information from peers via the WhatsApp messenger [25]. Likewise, another cross-sectional study was conducted in 2021 among Jordanian university students and involved different academic specialties [26]. The study found that students who study scientific- and medical-related majors tend to use their smartphones more than students studying humanities majors because medical students need to frequently access medical websites as well as communicate with peers for long hours to study in groups [26]. Based on the literature, smartphone overuse behavior among university students can be justified by their frequent use of phones for educational purposes.

Limitation

This study provides insight into the impact of smartphone overuse on academic performance among college students, and it is one of few research studies about the impact of smartphone overuse conducted in the Kingdom of Saudi Arabia. Nonetheless, it only focused on female students specializing in one field of study. Additionally, the sample size is small and the results can only be generalized to studies with similar settings. Furthermore, it didn’t examine the impact of smartphone use on the students’ social lives and psychological well-being. More studies among Saudi college students, both male and female, from different fields of studies are needed to examine the impact of smartphone overuse on different aspects of their lives. 

## Conclusions

The current study presented an insight into the smartphone overuse pattern among undergraduate nursing students and its impact on their academic achievement. The findings showed the impact of smartphone overuse on their academic achievement because smartphones have been used frequently for educational purposes. In conclusion, smartphones are useful devices that can be used effectively for different learning activities as they are shown to be accessible and affordable for most students.
